# Editorial: Computational neuroethology: simulating natural behaviors

**DOI:** 10.3389/fncom.2026.1971192

**Published:** 2026-09-14

**Authors:** Arij Daou, Angel A. Caputi

**Affiliations:** 1 Harvard Radcliffe Institute, Harvard University, Cambridge, MA, United States; 2 Neurophysiology and Computational Neuroscience Group, Biomedical Engineering Program, American University of Beirut, Beirut, Lebanon; 3 Sistema Nacional de Investigadores, Montevideo, Uruguay

**Keywords:** computational modeling, computational neuroethology, natural behavior, neural circuits, sensorimotor integration

Understanding how nervous systems generate adaptive behavior in natural environments remains one of neuroscience's central challenges. Much of what is known about neural function has necessarily come from experiments that simplify behavior, sensory input, and environmental structure. Yet animals do not normally act under such conditions. They navigate changing environments, exchange signals with conspecifics, pursue goals, and continuously modify their sensory input through movement. Computational neuroethology seeks to address this gap by combining ethological questions with quantitative models that can connect neural mechanisms to behavior while preserving the dynamics of the animal–body–environment system.

The six contributions to this Research Topic illustrate several complementary ways in which this goal can be pursued. Together, they span active sensing, social communication, sensorimotor control, neural-network simulation, and theoretical models of behavioral organization. Their common message is that computation is most powerful in neuroethology when it is not used only to describe behavior, but to formalize the mechanisms and constraints that make natural behavior possible.

This is especially clear in the three contributions centered on weakly electric fish. Pinto et al. present an open-source framework that combines multielectrode recordings, multi-camera video, deep-learning-based 3D tracking, and biophysical modeling of electric organ discharge signals in freely swimming *Gymnotus carapo*. By relating an animal's moment-to-moment position and posture to its self-generated electric field, this approach moves toward noninvasive reconstruction of behavior and sensory signaling under conditions in which conventional visual tracking may be difficult. Importantly, the framework also points toward closed-loop experiments in which computational estimates can guide stimulation during ongoing behavior.

Ramachandra et al. use spatially realistic modeling to ask how the distribution of electroreceptors and the geometry of the electric image constrain the detection and localization of conspecifics. Their results show that sensory performance cannot be inferred from receptor density alone: detection and angular resolution depend on the interaction among receptor organization, body geometry, signal geometry, and relative position. This provides a particularly clear example of why natural sensory computation must be understood at the level of the coupled animal–environment system.

Barayeu et al. similarly demonstrate the value of models when the behaviorally relevant stimulus space becomes too large for exhaustive electrophysiological sampling. Combining recordings with a model of electroreceptor afferents, they examine the encoding of communication chirps across social and sensory contexts. The work shows that chirps can remain detectable under high-frequency conditions that appear to lie outside classical receptor tuning, in part because frequency folding reshapes the effective electrosensory input. Here the model does more than reproduce data: it extends the experiment into otherwise difficult-to-sample regions of stimulus space and helps generate a mechanistic explanation for observations arising during natural social interactions.

Two further contributions emphasize the transformation from neural computation to action. Dickman et al. introduce NEURONpyxl, a Python-integrated framework for constructing and exploring conductance-based neural networks with complex synapses and plasticity. Using the feeding central pattern generator of *Aplysia*, they show how systematic parameter searches can identify network configurations that reproduce experimentally observed motor-pattern timing under different loading conditions. The framework is particularly relevant to computational neuroethology because it creates a path toward combining detailed neural circuitry with sensory feedback and biomechanics, enabling models to move from isolated neural dynamics toward embodied behavior.

Simmons et al. approach this problem from the perspective of active sensing. Their biomimetic SCAT biosonar model simulates a bat searching among multiple targets for a target with a specified acoustic structure. Target selection emerges through repeated cycles of sonar emission, echo analysis, beam orientation, movement, and renewed sampling. Although the simulated scenes deliberately omit several complexities of real acoustic environments, the study captures a defining feature of natural behavior: perception and action form a loop. The animal's movement changes subsequent sensory input, and sensory processing in turn guides the next action.

At a broader conceptual scale, Rahman et al. propose the ARCH × Φ framework, in which behavior emerges from interactions among archetypal systems, motivational drives, cultural context, and context-sensitive thresholds. Unlike the other contributions, this work is explicitly theoretical and remains only partially validated. Its relevance to the Research Topic lies in its attempt to formalize behavior as the outcome of converging internal and external constraints and, importantly, to translate that framework into falsifiable predictions. It therefore illustrates both the reach and the challenge of extending computational neuroethology toward increasingly complex forms of behavior.

Across these studies, an important principle emerges: natural behavior cannot be reduced to neural activity considered in isolation. The geometry of the body, the physics of sensory signals, environmental structure, social context, internal state, and the consequences of previous actions all shape the computations that nervous systems must perform. Computational models provide a common language for bringing these factors together. Their value is greatest when models and experiments operate iteratively—when natural behavior constrains the model, the model reveals hidden variables or generates predictions, and those predictions guide new measurements.

The next stage of computational neuroethology will require still tighter integration of high-resolution behavioral tracking, neural population recordings, realistic sensory environments, biomechanics, and closed-loop experimentation. Equally important will be open and reusable computational tools, transparent assumptions, and rigorous validation against behavior across contexts. The contributions assembled here show that this integration is already underway. Collectively, they move the field from computational description toward mechanistic, predictive, and increasingly embodied accounts of how nervous systems generate adaptive behavior in the real world.

